# Preparation, Optimisation, and In Vitro Evaluation of [^18^F]AlF-NOTA-Pamidronic Acid for Bone Imaging PET

**DOI:** 10.3390/molecules27227969

**Published:** 2022-11-17

**Authors:** Hishar Hassan, Muhamad Faiz Othman, Hairil Rashmizal Abdul Razak, Zainul Amiruddin Zakaria, Fathinul Fikri Ahmad Saad, Mohd Azuraidi Osman, Loh Hui Yi, Zarif Ashhar, Jaleezah Idris, Mohd Hamdi Noor Abdul Hamid, Zaitulhusna M. Safee

**Affiliations:** 1 Centre for Diagnostic Nuclear Imaging, Universiti Putra Malaysia (UPM), Serdang 43400, Malaysia; 2 Department of Pharmacy Practice, Faculty of Pharmacy, Universiti Teknologi MARA, Bandar Puncak Alam 42300, Malaysia; 3 Medical Imaging Program, Department of Health and Care Professions, Faculty of Health and Life Sciences, St Luke’s Campus, University of Exeter, Devon EX1 2LU, UK; 4 Borneo Research on Algesia, Inflammation and Neurodegeneration (BRAIN) Group, Department of Biomedicine, Faculty of Medicine and Health Sciences, Universiti Malaysia Sabah, Kota Kinabalu 88400, Malaysia; 5 Department of Cell and Molecular Biology, Faculty of Biotechnology and Biomolecular Sciences, Universiti Putra Malaysia (UPM), Serdang 43400, Malaysia; 6 Department of Nuclear Medicine, National Cancer Institute, Putrajaya 62250, Malaysia

**Keywords:** ^18^F, aluminium fluoride (Al-F), pamidronic acid, radiopharmaceuticals, radiochemistry, positron emission tomography, bone imaging

## Abstract

[^18^F]sodium fluoride ([^18^F]NaF) is recognised to be superior to [^99^mTc]-methyl diphosphate ([^99m^Tc]Tc-MDP) and *2-deoxy-2-[^18^F]fluoro-D-glucose* ([^18^F]FDG) in bone imaging. However, there is concern that [^18^F]NaF uptake is not cancer-specific, leading to a higher number of false-positive interpretations. Therefore, in this work, [^18^F]AlF-NOTA-pamidronic acid was prepared, optimised, and tested for its in vitro uptake. NOTA-pamidronic acid was prepared by an *N-*Hydroxysuccinimide (NHS) ester strategy and validated by liquid chromatography-mass spectrometry analysis (LC-MS/MS). Radiolabeling of [^18^F]AlF-NOTA-pamidronic acid was optimised, and it was ensured that all quality control analysis requirements for the radiopharmaceuticals were met prior to the in vitro cell uptake studies. NOTA-pamidronic acid was successfully prepared and radiolabeled with ^18^F. The radiolabel was prepared in a 1:1 molar ratio of aluminium chloride (AlCl_3_) to NOTA-pamidronic acid and heated at 100 °C for 15 min in the presence of 50% ethanol (*v*/*v*), which proved to be optimal. The preliminary in vitro results of the binding of the hydroxyapatite showed that [^18^F]AlF-NOTA-pamidronic acid was as sensitive as [^18^F]sodium fluoride ([^18^F]NaF). Normal human osteoblast cell lines (hFOB 1.19) and human osteosarcoma cell lines (Saos-2) were used for the in vitro cellular uptake studies. It was found that [^18^F]NaF was higher in both cell lines, but [^18^F]AlF-NOTA-pamidronic acid showed promising cellular uptake in Saos-2. The preliminary results suggest that further preclinical studies of [^18^F]AlF-NOTA-pamidronic acid are needed before it is transferred to clinical research.

## 1. Introduction

Primary bone cancer is defined as cancer that originates in the bone itself. It can be benign (non-cancerous) or malignant (cancerous), the latter being less common than benign primary bone cancer [1]. However, both types of primary bone cancer are capable of growing and compressing healthy bone tissue. Bone metastases occur when cancer cells from primary cancer migrate into the bone. Osteosarcoma is a classic primary bone cancer characterised by the presence of malignant mesenchymal cells in the bone stroma [2]. The cancer is primary when it is localised, and there is no evidence that the malignant cells have spread outside the bone, and it is considered secondary (metastatic) when it has spread to distant parts of the body [3]. Cancer Research UK reported a survival rate of 40% for people with osteosarcoma who survived their cancer for 5 years among the population in England, while the American Cancer Society reported an average survival rate of 70% [4,5].

On the other hand, bone metastases are a common feature in patients with advanced prostate cancer, breast cancer, and lung cancer and remain the leading cause of death in advanced prostate cancer [6,7]. Bone metastases lead to complications such as severe pain, bone fractures, spinal cord compression, and bone marrow suppression [8,9]. Under these circumstances, the early detection of primary bone cancer and bone metastases is crucial for the prevention of skeletal-related events. For bone diagnostics, in particular, several imaging techniques have been investigated and compared in terms of their sensitivity and specificity [10]. European guidelines recommend cost-effective single-photon emission computed tomography (SPECT) using [^99m^Tc]-methyl diphosphonate ([^99m^Tc]Tc-MDP) for bone diagnosis. However, SPECT imaging has some weaknesses, particularly in quantifying the response to treatment [11]. A major drawback worth highlighting is the slow distribution and excretion of [^99m^Tc]Tc-MDP, possibly due to the direct complexation of the radiometal [^99m^Tc] and methyl diphosphonate (MDP) in the [^99m^Tc]Tc-MDP complex. Furthermore, its specificity is limited as uptake is also observed in non-cancer cells [12,13]. This phenomenon could represent an increased risk for the under-staging and under-treatment of the disease [14]. Meanwhile, positron emission tomography (PET) has relied on *2-deoxy-2-[^18^F]fluoro-D-glucose* (2-[^18^F]FDG) for most oncological cases. However, 2-[^18^F]FDG uptake was variable in blastic lesions, and cranial bone involvement was reportedly overlooked due to physiological brain metabolism [15]. By contrast, a new marker targeting C-X-C chemokine receptor type 4 (CXCR-4) expression, [^68^Ga]Ga-Pentixafor, appears to be suitable only for the diagnosis of the chronic infection of the bone [16].

A multicentre study concluded that [^18^F]sodium fluoride ([^18^F]NaF) is superior to [^99m^Tc]Tc-MDP and 2-[^18^F]FDG in the diagnosis of bone metastases [15,17,18]. In this study, all [^18^F]NaF PET/CT and [^99m^Tc]Tc-MDP scans were positive for bone metastases, while 2-[^18^F]FDG results were negative in some cases. Further cross-sectional imaging proved that [^18^F]NaF PET/CT was advantageous over [^99m^Tc]Tc-MDP scans, as some lesions that were missed on [^99m^Tc]Tc-MDP scans were detected by [^18^F]NaF PET/CT [15]. Nevertheless, there is concern that [^18^F]NaF uptake is not cancer-specific, leading to a higher number of false-positive interpretations [19]. Currently, there are several efforts to identify early markers for bone imaging and new drug targets to improve the quality of life for patients with skeletal-related events caused by bone metastases. Therefore, there is a need for accurate imaging, proper staging, and assessment of the response to treatment and long-term oncological management, as this is directly related to patient morbidity and healthcare costs [19].

Bisphosphonates (BPs) are a group of drugs that were discovered back in the 1960s. They bind strongly to bone minerals, which gives them the unique property of selective uptake [20,21]. BPs bind strongly to bone minerals via calcium coordination in the hydroxyapatite lattice, which differs from the binding of fluoride in that it displaces the hydroxide in the hydroxyapatite lattice and converts it to fluorapatite [22,23]. Over the years, modifications to the structure of BPs have been studied to improve their pharmacodynamic behaviour, mainly to increase their bone-binding affinity. BPs are divided into two main groups: nitrogen-containing BPs (N-BPs) and non-nitrogen-containing BPs. Comparative studies have shown that the N-BPs form additional hydrogen bonds and have a higher bone-binding affinity [21]. The N-BPs include pamidronic acid, zoledronic acid, risedronic acid, alendronic acid, and ibandronic acid. Previous studies concluded that pamidronic acid has the highest bone-binding affinity, followed by alendronic acid, zoledronic acid, risedronic acid, and finally, ibandronic acid [24,25].

In recent years, the number of targeted radiopharmaceutical markers for bone imaging has increased. For example, recent developments using ^68^Ga-labeled bisphosphonates have been investigated for PET bone imaging [26,27,28]. However, there has been insufficient innovation in ^18^F radiopharmaceutical derivatives for bone imaging. Therefore, there is a need to develop an ^18^F radiopharmaceutical derivative that can be used more specifically for PET bone imaging. ^18^F can be produced in high yield in a modern medical cyclotron compared to ^68^Ga produced by generators with a limited activity per elution. Another important aspect is that ^18^F has a longer half-life, so it can be transported to remote hospitals without a cyclotron on site. One of the most important issues is that approved ^68^Ge/^68^Ga generators have become very expensive and have long delivery times, which further weakens the rationale for ^68^Ga radiopharmaceuticals [29]. Therefore, replacing ^68^Ga with ^18^F can lead to a reliable supply with a significant cost reduction [30]. The formation of ^18^F linked to the bifunctional chelating agent was laborious until McBride et al., in 2009, developed an excellent method that exploited the fluorophilic nature of aluminium to allow its direct complexation with [^18^F]F^−^ and to form stable aluminium fluoride complexes ([^18^F]AlF^2+^) [31]. A bifunctional chelating agent can be covalently attached to a peptide, small protein, or compound of interest and seamlessly coordinated with [^18^F]AlF^2+^ complexes using this method. This novel approach leads to shorter reaction times, more efficient radiochemistry, and a better economic approach. It enables radiolabeling in aqueous media through a one-pot reaction [30,32,33]. Furthermore, the method also solves the problem associated with ^68^Ga complexation and classical carbon-^18^F radiochemistry [34]. Therefore, it is now possible to label various targeting vectors that were previously labelled with the radiometal ^68^Ga or other radionuclides with ^18^F in a high yield without having to invest in an expensive ^68^Ge/^68^Ga generator which a radiopharmacy centre that does not have one [35].

To find out whether ^18^F-labeled bisphosphonate has the same problem as [^18^F]NaF in terms of non-cancer uptake, the present work aims to produce a new targeting vector by first forming the [^18^F]AlF^2+^, followed by coordinating the positively charged [^18^F]AlF^2+^ to the NOTA-pamidronic acid moiety and optimising the radiolabeling of [^18^F]AlF-NOTA-pamidronic acid. Pamidronic acid was selected as a targeting vector for bone imaging because it has the highest bone-binding affinity compared to other bisphosphonates, as reported by Jahnke et al. [25]. Ultimately, this study also aims to demonstrate the cellular uptake of [^18^F]AlF-NOTA-pamidronic acid through an in vitro study using normal human osteoblast cell lines (hFOB 1.19) and human osteosarcoma cell lines (Saos-2) to demonstrate the concept that [^18^F]AlF-NOTA-pamidronic acid can potentially be used for PET bone imaging.

## 2. Results and Discussion

### 2.1. Preparation, Validation, and Isolation of NOTA-Pamidronic Acid

#### 2.1.1. Preparation of NOTA-Pamidronic Acid

**Figure 1:**
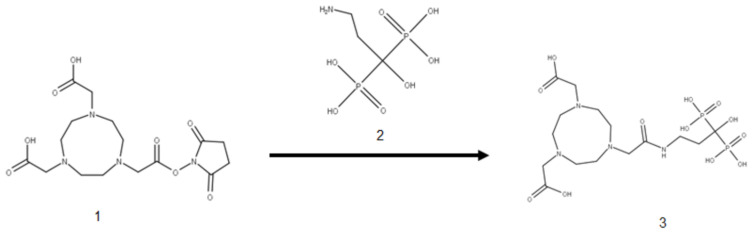
Conjugation of NOTA-NHS (1) and pamidronic acid (2) for the formation of NOTA-pamidronic acid (3).

The preparation of the NOTA-pamidronic acid (Figure 1) involved the conjugation of NOTA chelator with pamidronic acid, which acted as a vector molecule, using the *N-*hydroxysuccinimide (NHS) ester strategy. The NOTA-NHS ester-activate reacted with the primary amines of the pamidronic acid to form stable amide bonds while NHS was released. The primary amine group (NH_2_) was known to be an easy target for conjugation [36]. The conjugation was carried out at room temperature for 4 h by adding a NOTA-NHS ester to a pamidronic acid solution adjusted to pH 8 with TEA [37,38].

**Figure 2:**
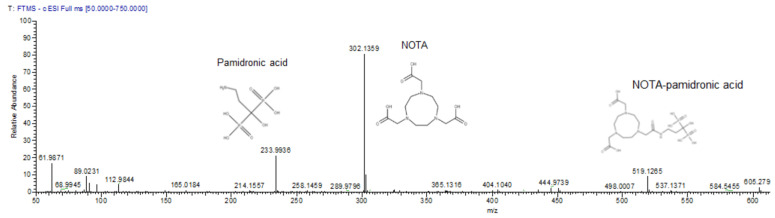
The mass spectrum of pamidronic acid, NOTA-pamidronic acid, and free NOTA producing *m*/*z* [M-H] of 234, 302, and 519 respectively.

Although NOTA-NHS was prepared by dissolving in dimethylformamide (DMF), there was a possibility that hydrolysis might occur during the reaction due to the presence of water from the pamidronic acid solution. Consequently, the unconjugated NOTA-NHS was hydrolysed to form free NOTA. Therefore, the mass spectrometric analysis also detected the free NOTA (fragment) with an exact molecular weight of 302.136 g mol^−1^ (Figure 2).

A major reason for dissolving the NOTA-NHS ester chelator in an organic solvent, such as DMF in this case, is that the NHS esters in the NOTA-chelator are relatively insoluble in water and must first be dissolved in an organic solvent [39]. Furthermore, when a compound containing an NHS ester is dissolved in water, it immediately begins to hydrolyse, which can reduce the yield of the NOTA-pamidronic acid [40]. The conditions (pH 8 and room temperature) for this reaction appeared to be sufficient to allow for conjugation. In general, a reasonable physiological to basic pH was sufficient for the reaction to take place.

#### 2.1.2. Validation of NOTA-Pamidronic Acid Using LC-MS Analysis

**Figure 3:**
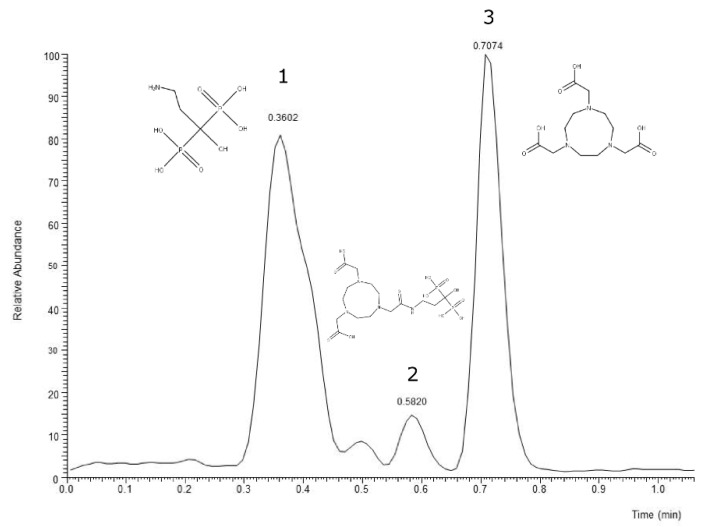
LC-MS chromatogram of crude NOTA-pamidronic acid sample. (1) pamidronic acid; (2) NOTA-pamidronic acid; (3) free NOTA.

The validation of NOTA-pamidronic acid from conjugation was performed using liquid chromatography-mass spectrometry (LC-MS) analysis. Liquid chromatography conditions for mass spectrometry analysis were performed according to the ion suppression reversed-phase chromatography. Figure 3 shows that pamidronic acid (m.w. 233.9936), NOTA-pamidronic acid (m.w. 519.1269), and free NOTA (m.w. 302.1359) eluted at the retention times (Rt) of 0.36, 0.58, and 0.71 min, respectively. The result of the chromatogram in Figure 2 confirms that the difference in polarity between these three compounds, with pamidronic acid being the most polar and free NOTA being the least polar, results in the pamidronic acid eluting first, followed by NOTA-pamidronic acid and free NOTA last.

With a molar ratio of 5:1 (Pamidronic acid: NOTA-NHS), a yield of 24.13% was obtained. The figure explains how about 24.13% of the pamidronic acid with an initial weight of 5.875 mg was theoretically converted into 1.418 mg of NOTA-pamidronic acid.

Table 1 shows how as the concentration of pamidronic acid increased, the amount of NOTA-pamidronic acid also increased. This shows that there was a strong relationship between the percentage of NOTA-pamidronic acid yield and the ratio of pamidronic acid: NOTA-NHS. This supports Hermanson et al., who identified an optimised product yield when the ratio of target molecules to the NHS crosslinker was increased [39]. When the concentration of pamidronic acid (the target molecule) was increased, the amount of NOTA-NHS coupled to the pamidronic acid certainly increased. Unfortunately, the amount of non-conjugated pamidronic acid (free pamidronic acid) also increased, as observed in the peak area for non-conjugated pamidronic acid. This resulted in a decrease in the percentage yield of NOTA-pamidronic when pamidronic acid was included in the calculation of the percentage yield of NOTA-pamidronic acid.

A statistical analysis of one-way ANOVA was performed and confirmed that there was a statistical difference between the percentage yield of NOTA-pamidronic acid when the molar ratio of pamidronic acid and NOTA was varied (*p* < 0.05). Furthermore, Bonferroni posthoc analysis was performed to determine the molar ratio of the pamidronic acid to NOTA-NHS, which demonstrated a significant difference in the percentage yield of NOTA-pamidronic acid. The Bonferroni post hoc analysis revealed that changing the molar ratio of pamidronic acid to NOTA-NHS produced a significant difference in the percentage yield of NOTA-pamidronic acid across the group.

Another viable alternative to maximise the amount of NOTA-pamidronic acid produced in the reaction was to increase the molar excess of NOTA-NHS over the pamidronic acid. However, an excess of the NOTA compound (free NOTA) would remain, potentially competing with the NOTA-pamidronic acid for [^18^F]AlF^2+^ coordination during radiolabeling. Therefore, the purification of the NOTA-pamidronic acid crude sample is essential to remove the free NOTA as much as possible from the crude sample.

The possibility of increasing the amount of NOTA-pamidronic acid precursor produced in the reaction was also explored by using dimethyl sulfoxide (DMSO) as an organic solvent, which is more polar than DMF. The experiment was repeated with DMSO to dissolve the NOTA-NHS before pamidronic acid was added at a molar ratio of 5:1. However, the yield of the NOTA-pamidronic acid preparation was only highest at 12.95% when DMSO was used. A statistical analysis of the independent samples *t*-test was performed and confirmed that there was a statistical difference between the percentage yield of NOTA-pamidronic acid when using DMF and DMSO as an organic solvent (*p* < 0.05). An interesting point about using DMSO was that it was more difficult to eliminate DMSO during the post-reaction than DMF. This is consistent with the findings of other researchers who have also used DMSO as an organic solvent, as DMSO takes longer to dry and is more difficult to eliminate [40]. The study, therefore, recommends using DMF as an organic solvent during conjugation.

#### 2.1.3. Mass Spectrometry Analysis of NOTA-Pamidronic Acid

The important piece of information from the mass spectrometry analysis was the mass-to-charge ratio (*m*/*z*) value of the conjugated product. The *m*/*z* value of the conjugated product is crucial to determine whether the preparation of the NOTA-pamidronic acid precursor was successful or not. Most importantly, it can be difficult to independently characterise the conjugated product using analytical liquid chromatography alone, as there is no non-commercially available NOTA-pamidronic acid reference standard.

Since the ionisation mode of electrospray ionisation (ESI) in this experiment was set to a negative mode, the *m*/*z* value of the NOTA-pamidronic acid ion was one proton lower due to the abstraction of a proton [M-H]^−^. The negative ion mode was preferred over the positive ion mode in this experiment because of the presence of carboxyl groups [41]. Therefore, the precursor ion was expected to be [M-H]^−^ due to deprotonation from the molecular formula.

Table 2 shows that the [M-H]^−^ *m*/*z* values for pamidronic acid (A), NOTA-pamidronic acid (B), and free NOTA (C) were 233.9934, 519.1265, and 302.1358, respectively. The relative error of the obtained *m*/*z* values was less than 1 ppm. The relative error for all three compounds was below the acceptable limit of between 2 and 5 ppm, especially for an Orbitrap mass analyser [42].

The evidence confirmed that the samples prepared from the conjugation of pamidronic acid and NOTA-NHS, yielded a NOTA-pamidronic acid precursor, as the obtained *m*/*z* (m.w. 519.1265) was similar to the calculated *m*/*z* (m.w. 519.1263) (Figure 2).

#### 2.1.4. Fragmentation Analysis of NOTA-Pamidronic Acid

Another important piece of information obtained from the MS–MS analysis was the fragment ions. In the MS–MS analysis, the selected precursor ions of NOTA-pamidronic acid (m.w. 519.1265) were broken down into fragments (product ions). In this study, the MS–MS analysis was used to ensure that the conjugated sample was NOTA-pamidronic acid. For this purpose, the spectral analysis of the NOTA-pamidronic acid fragment (product) ions was compared with the predicted spectra of the fragment ions obtained from the competitive fragmentation modelling for metabolite identification (CFM-ID) (http://cfmid.wishartlab.com, accessed on 25 July 2022) (Table 3).

The elemental composition for 519 was determined to be 15 carbon (C), 29 hydrogens (H), 4 nitrogens (N), 12 oxygens (O), and 2 phosphorus (P). Table 3 shows the ring plus double bond equivalence (RDBE) of the NOTA-pamidronic acid precursor ion (C_15_H_29_N_4_O_12_P_2_) was 4.5. This shows that the structure of NOTA-pamidronic acid contains one ring and three double bonds (carbonyl). The value of the RDBE of NOTA-pamidronic acid shows that this precursor ion is an even-electron ion (EE), which is consistent with the nitrogen rule (N Rule) that an odd-numbered precursor ion (RDBE value of 4.5) would have an even number of nitrogens (4 nitrogens) for an EE.

**Figure 4:**
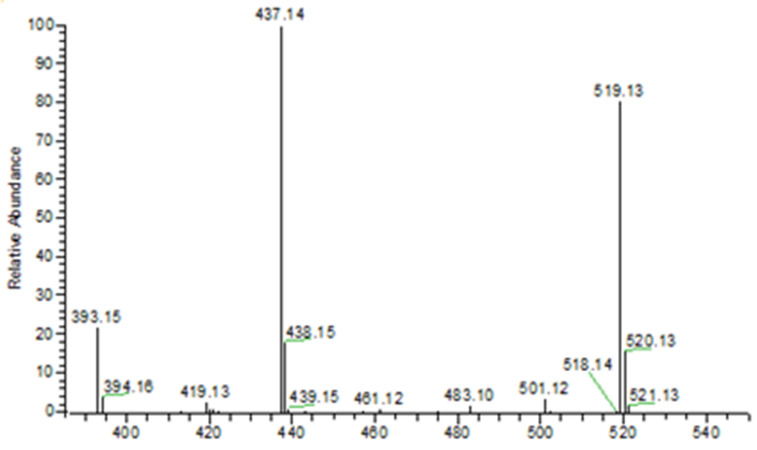
ESI (- negative) mass spectrum of NOTA-pamidronic acid producing 519 [M-H]^−^ precursor ion and 437 [M-H-H_3_PO_3_]^−^ base peak.

**Figure 5:**
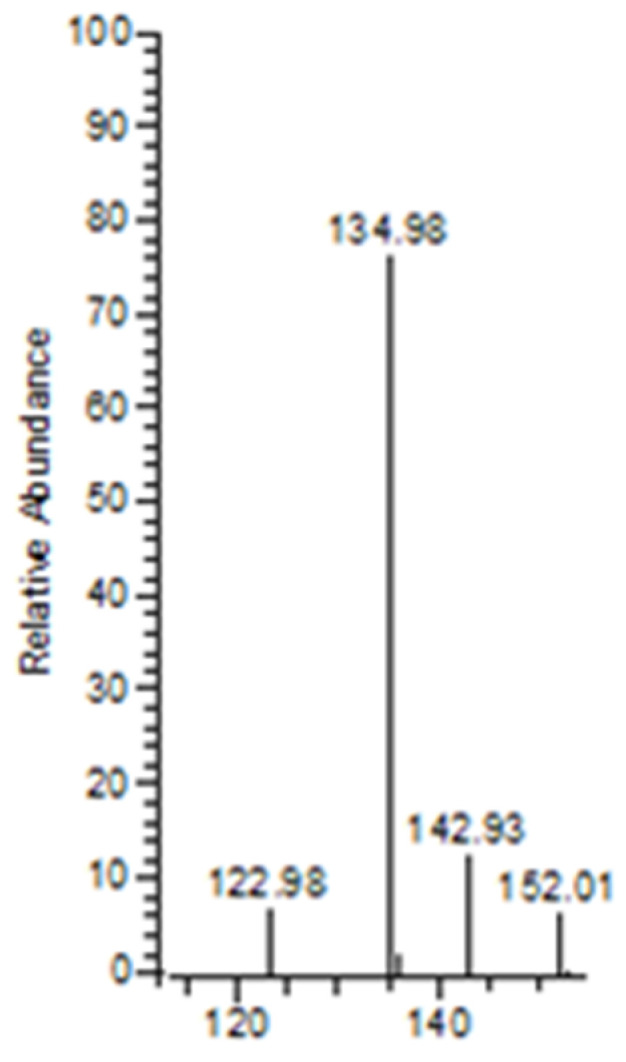
Diagnostic ions for BPs were observed at *m*/*z* 135, 143, and 152. The ion observed at *m*/*z* 143 indicates the presence of a BP group, such as pamidronic acid in this case.

The neutral loss observed in the MS–MS analysis was 18, 64, 82, and 44, derived from H_2_O, HPO_2_, H_3_PO_3,_ and CO_2_, respectively. Based on the fragments generated (Figure 4), the primary fragment ions were *m*/*z* 501 [M-H-H_2_O]^−^, 437 [M-H-H_2_O-HPO_2_]^−^/[M-H-H_3_PO_3_]^−^, and 393 [M-H-CO_2_]^−^/[M-H-H_3_PO_3_-CO_2_]^−^. The ESI-MS-MS produced two fragment ions, *m*/*z* 501 and 437, through the neutral loss of H_2_O and H_3_PO_3_, respectively. The *m*/*z* 501 was produced by the -OH dehydration of two phosphorus groups forming a four-membered ring. The fragment ion of *m*/*z* 437 resembled [M-H-H_3_PO_3_]^−^. The ESI-MS-MS of *m*/*z* 437 produced three fragment ions, observed at *m*/*z* 393, 152, and 135. The *m*/*z* 393 was produced by the neutral loss of carbon dioxide [M-H-H_3_PO_3_-CO_2_]^−^. The ESI-MS-MS of *m*/*z* 501 produced a fragment ion of 143. The *m*/*z* 143 in the lower mass series was found to be an identical fragment ion for the compounds with a bisphosphonate group (Figure 5). Figure 4 shows that the base peak was at an *m*/*z* value of 437.

**Figure 6:**
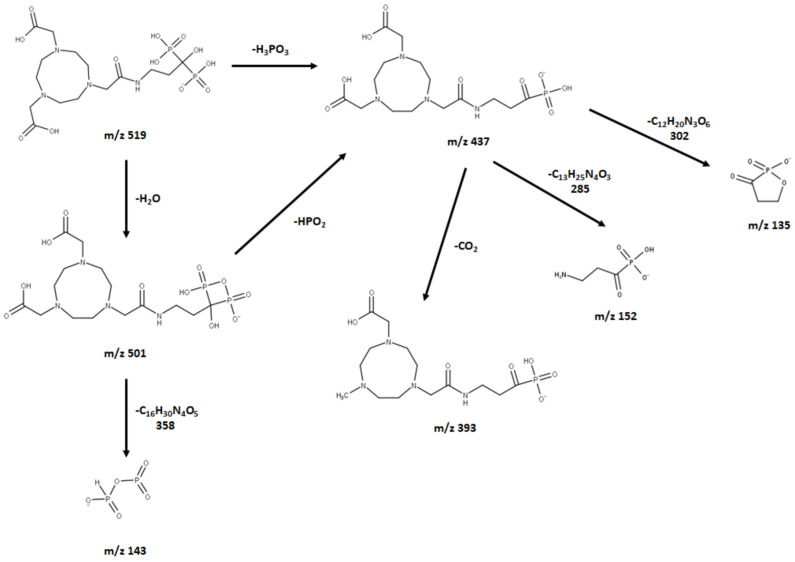
Proposed (−) ESI-MS fragmentation of NOTE-pamidronic acid.

The observed base peak with the *m*/*z* value of 437, corresponds to two possible pathways (Figure 6) for the production of the fragment ion *m*/*z* 437: [M-H-H_2_O-HPO_2_]^−^/[M-H-H_3_PO_3_]^−^ [43]. The abundance of the fragment ion *m*/*z* 437 was due to the stability and low proton affinity of the neutral loss (Field’s rule). The increase in the unsaturation of *m*/*z* 437 based on an RDBE value of 5.5 reflects this increased stability.

The *m*/*z* 501 and 437 fragment ions produced characteristic ions in a lower mass series. Three fragment ions with *m*/*z* values of 135, 143, and 152 were observed in the lower mass series (Figure 5). The characteristic ion observed, in particular, at an *m*/*z* of 143 indicates the presence of a BP group, such as pamidronic acid, in this case, which is consistent with other findings [43]. The fragment ions produced are consistent with the EE rule, which favours the heterolytic process via the charge retention fragmentation (CRF) pathway [44].

#### 2.1.5. Isolation of the NOTA-Pamidronic Acid Fraction from the Crude Sample

**Figure 7:**
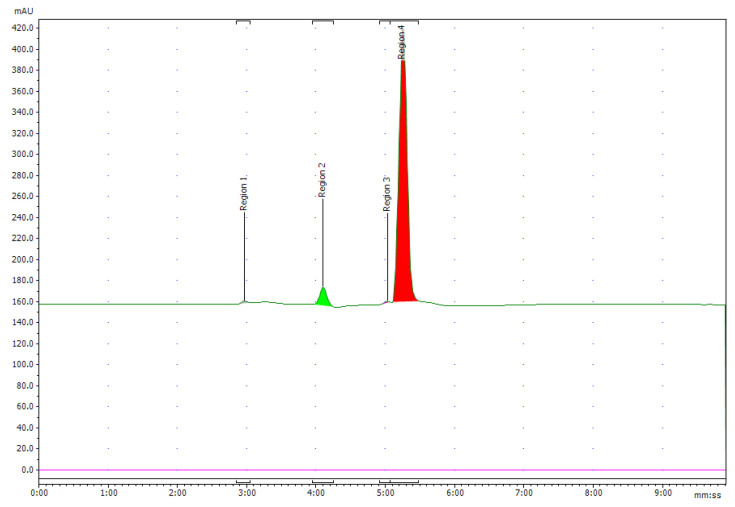
Chromatogram of UV 220 nm of pure NOTA-pamidronic acid post isolation.

The peak corresponding to NOTA-pamidronic acid (Rt: 5.28 min, *SD* = 0.24 min) was collected using reverse-phase high-performance liquid chromatography (RP-HPLC) (Agilent 1200, USA) equipped with a fraction collector. The isolation of the NOTA-pamidronic acid fractions was carried out under optimal chromatographic conditions as described in Section 3.2.3. The collected NOTA-pamidronic acid fractions were re-analysed using the analytical method RP-HPLC. The purity of the NOTA-pamidronic acid was determined by the peak area of the isolated NOTA-pamidronic acid fractions compared to other peaks of the RP-HPLC chromatogram. Figure 7 below shows the chromatogram of the NOTA-PAM fractions in which NOTA-pamidronic acid was detected at the highest peak of number 4 (Rt: 5.15 min). This shows that the collected NOTA-pamidronic acid fractions had the highest purity. Peak numbers 1, 2, and 3 were detected as unknown impurities. Free NOTA was not detectable in the isolated NOTA-pamidronic acid fractions because the peak at an Rt of 7.56 min (*SD* = 0.08), corresponding to free NOTA, was not present.

The purity of the isolated NOTA-pamidronic acid fractions was 92.2% (*SD* = 1.9, *n* = 3) with an observed molecular mass of 519.1265 ± 0.0004 (theoretical molecular mass: 519.1263). The collected NOTA-pamidronic acid fractions were then sent for freeze-drying to improve their stability and prevent the possible degradation of the final product by removing water from the final product. The freeze-dried NOTA-pamidronic acid was then stored in the freezer at −20 °C.

### 2.2. Optimisation of [^18^F]AlF-NOTA-Pamidronic Acid Radiolabeling Conditions

The optimisation of the [^18^F]AlF-NOTA-pamidronic acid radiolabeling conditions was carried out in two stages. In the first stage, the optimisation of the radiolabeling conditions for [^18^F]AlF^2+^ complexation was evaluated by examining the AlCl_3_ concentration (3.2.4.1. the preparation of [^18^F]AlF^2+^ complexes). The RCY of the [^18^F]AlF^2+^ complexes was determined from an aliquot of the reaction solution and evaluated with a Sep-Pak cartridge combination. In the second stage, the radiolabeling conditions for the [^18^F]AlF-NOTA complexation were then optimised using a non-conjugated NOTA-NHS chelator before using a NOTA-pamidronic acid precursor. In attempting to optimise the radiolabeling conditions for the [^18^F]AlF-NOTA complexation, four variables were identified: (1) the molar ratio of AlCl_3_ to the NOTA-pamidronic acid, (2) the reaction time, (3) the reaction temperature, and (4) the co-solvent that would potentially affect the formation of [^18^F]AlF-NOTA-pamidronic acid complexes.

#### 2.2.1. [^18^F]F^−^ Activity

About 83–95% of the ^18^F radioactivity was eluted from fractions two to three. The fractionation technique was able to concentrate 113 MBq of [^18^F]F^−^ in a volume of 200 μL and minimise contamination (with metallic impurities) due to the smaller volume of the eluate. Some short-lived radionuclides, such as nitrogen-13 (^13^N) and oxygen-15 (^15^O), were among the most likely non-metallic radionuclide contaminants in the aqueous ^18^F solution. However, both have extremely short half-lives, of about 10 min and 2 min for ^13^N and ^15^O, respectively, and would decay naturally during transport (in our case) or before radiolabeling began.

The potential ^18^F contaminants varied depending on the target system used in the cyclotron. A niobium target with a Havar foil released mainly manganese (Mn), cobalt (Co), and technetium (Tc) species, which are normally trapped in the Sep-Pak QMA cartridge [45]. This shows that impurities in an aqueous ^18^F solution freshly produced from a cyclotron can be removed by solid phase extraction and fractionation before using the aluminium-fluoride (Al-F) technique [46].

Preliminary results showed that the formation of [^18^F]AlF^2+^ was significantly lower when the solid phase extraction and fractional elution were not performed. It was suggested that this was due to other ionic impurities in the aqueous ^18^F solutions, such as iron (II) ion (Fe^2+^), copper (II) ion (Cu^2+^), zinc (II) ion (Zn^2+^), ammonium (NH^4+^), which could compete with Al^3+^ in the formation of [^18^F]AlF^2+^. However, the evaluation of radionuclide ^18^F in the aqueous solution was not considered in this study. Therefore, it was recommended to perform the solid phase extraction (SPE) and fractional elution and to use the highest fractional [^18^F]F^−^ activity for the formation of [^18^F]AlF^2+^.

#### 2.2.2. Effect of AlCl_3_ Concentration on the Radiochemical Yield (RCY) of [^18^F]AlF^2+^ Complexes

[^18^F]AlF^2+^ complexes were formed by the reaction of AlCl_3_ with [^18^F]F^−^ anions in an aqueous solution between pH 4 and 5. Although 20 mM AlCl_3_ resulted in the highest formation of [^18^F]AlF^2+^ complexes, 99.94% (*SD* = 0.1, *n* = 3), further statistical analysis of the independent *t*-test revealed that increasing the AlCl_3_ concentration did not significantly increase the RCY of the [^18^F]AlF^2+^ complexes (*p* > 0.05). The difference in RCY of the [^18^F]AlF^2+^ complexes was too small. While 5 and 20 mM AlCl_3_ resulted in higher [^18^F]AlF^2+^ complexes (*M* = 99.92%, *SD* = 0.1 for 5 mM), higher amounts of AlCl_3_ were required to achieve the exact complexation yield with 2 mM AlCl_3_ (*M* = 99.88%, *SD* = 0.2, *n* = 3). Since the results show that a lower concentration of AlCl_3_ (2 mM) can produce comparable [^18^F]AlF^2+^ complexes to a higher concentration of AlCl_3_ (5 mM and 20 mM), the use of AlCl_3_ at a lower concentration is, therefore, more optimal. Therefore, 2 mM AlCl_3_ was used for the [^18^F]AlF^2+^ complexation.

#### 2.2.3. Effect of Reaction Temperature and Time on the Formation of the [^18^F]AlF-NOTA-NHS Complex

The radiolabeling conditions were first optimised using a complexation assay with the inexpensive NOTA NHS chelator. Radiolabeling conditions were set at three different temperatures (60, 80, and 100 °C) and reaction times (5, 10, and 15 min) to investigate the effects of the reaction temperature and time on the RCY of the [^18^F]AlF-NOTA-NHS complex. The RCY of the [^18^F]AlF-NOTA NHS complex was proportional to the increase in the reaction temperature. The [^18^F]AlF-NOTA NHS complex had the highest RCY, 94.6% (*SD* 0.9) when the reaction temperature and time was 100 °C for 15 min and the lowest when the reaction temperature was 60 °C (*M* = 48.5%, *SD* = 36.9). These results were consistent as the NOTA chelator needed to be heated to 100–120 °C [47]. Since the NOTA chelator is cyclic, the activation energies for chelating the metal ions are significantly higher than for the linear chelator [48]. To overcome these considerable kinetic barriers in the radiolabeling of NOTA conjugates with [^18^F]AlF^2+^, reaction solutions were heated to 100–120 °C [48].

Nevertheless, it has been pointed out that these reaction conditions are problematic when the chelator is conjugated with heat-sensitive biomolecules [47,49]. Further statistical analysis of the one-way ANOVA revealed that the reaction time between 5 and 15 min had no significant effect on the RCY of the [^18^F]AlF-NOTA NHS complex when heated at 100 °C (*p* > 0.05). The RCY of the [^18^F]AlF-NOTA NHS complex was above 90% in all experiments with reaction times. Therefore, a viable strategy for a chelator conjugated to heat-sensitive biomolecules is a short reaction time: between 5 and 10 min at 100 °C.

#### 2.2.4. Effect of Organic Solvent and Percentage of Organic Solvent

**Figure 8:**
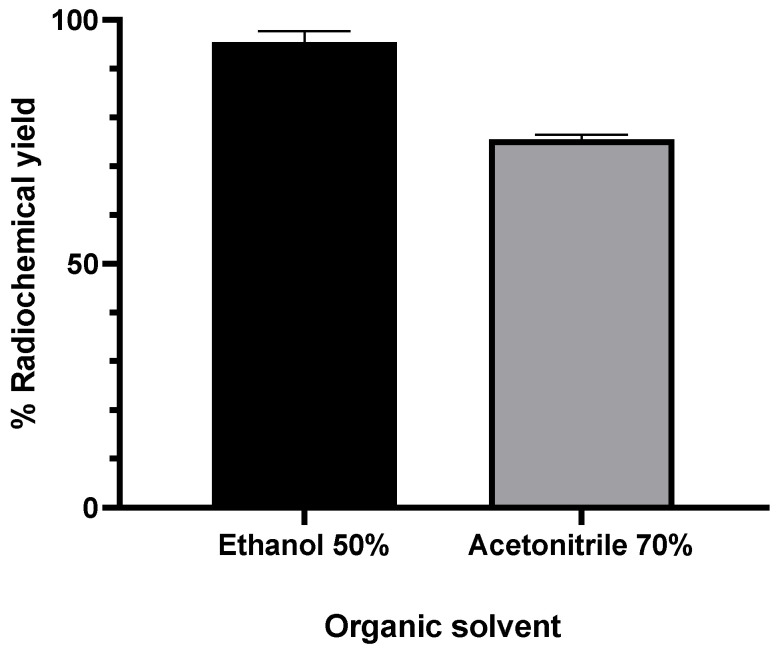
RCY of [^18^F]AlF-NOTA-pamidronic acid when different organic solvents were added to the reaction mixture (*n* = 6); mean (SEM).

Previous results showed that the formation of the [^18^F]AlF-NOTA NHS complex was first and second highest when acetonitrile 70% (*v*/*v*) or ethanol 50% (*v*/*v*) were added as organic solvents [50]. To ensure reproducibility, the radiolabeling conditions of the NOTA-pamidronic acid precursor were repeated using acetonitrile 70% (*v*/*v*) and ethanol 50% (*v*/*v*) as organic solvents. The formation of [^18^F]AlF-NOTA-pamidronic acid from both organic solvents was determined by the radio-thin layer chromatography technique (r-TLC). In contrast to the previous results, the formation of [^18^F]AlF-NOTA-pamidronic acid was highest (*M* = 95.50%, *SD* = 5.34) when ethanol 50% (*v*/*v*) was used as an organic solvent (*n* = 6) (Figure 8). The formation of [^18^F]AlF-NOTA-pamidronic acid, determined by r-TLC, was only 75.55% (*SD* = 2.21, *n* = 6) when acetonitrile 70% (*v*/*v*) was added to the reaction mixture (Figure 8).

Further statistical analysis of the independent samples *t*-test showed that the difference in the formation of [^18^F]AlF-NOTA-pamidronic acid was significant when ethanol 50% (*v*/*v*) was added to the reaction mixture (*p* < 0.05). There is a possible explanation that could justify the higher formation of [^18^F]AlF-NOTA-pamidronic acid when ethanol was used compared to acetonitrile. The presence of ethanol facilitates the interaction of metal cations, [^18^F]AlF^2+^, with donors in the chelate structure, such as, in this case, NOTA-pamidronic acid moiety and, therefore, leads to a higher forms of [^18^F]AlF-NOTA-pamidronic acid [38].

The results showed that the use of acetonitrile was only effective in coordinating [^18^F]F^−^ with the Al^3+^ to form [^18^F]AlF^2+^ in the previous suggestion. One could speculate that the difference in the structure of NOTA-pamidronic acid compared to NOTA-NHS could contribute to the higher formation of [^18^F]AlF-NOTA-pamidronic acid when ethanol 50% (*v*/*v*) was used. Since the formation of [^18^F]AlF-NOTA-pamidronic acid was higher when ethanol 50% (*v*/*v*) was used, ethanol was chosen as the organic solvent. Furthermore, ethanol was the most biocompatible of all the solvents [51].

#### 2.2.5. The Optimal Ratio between AlCl_3_ and NOTA-Pamidronic Acid

**Figure 9:**
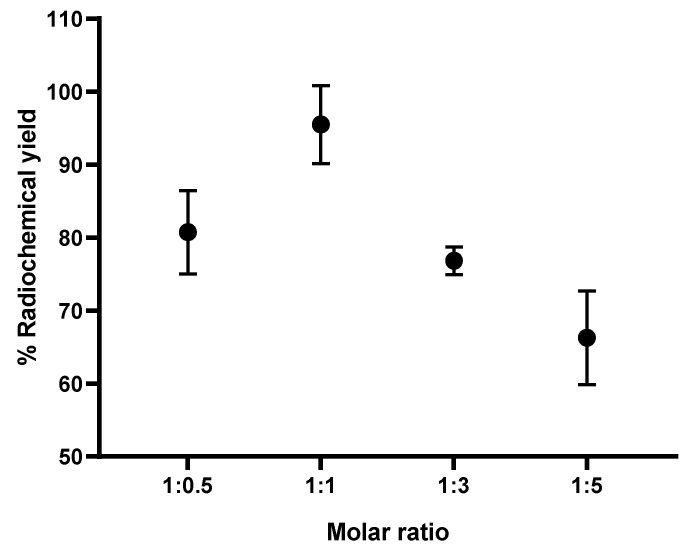
RCY of [^18^F]AlF-NOTA-pamidronic acid (*n* = 6).

Based on the preliminary results [50], the formation of the [^18^F]AlF-NOTA NHS complex was above 80% for all AlCl_3_-to-NOTA molar ratios prepared. Nevertheless, a statistical analysis of one-way ANOVA revealed that the difference in the percentage of the RCY (formation) of the [^18^F]AlF-NOTA NHS complex between the prepared molar ratios of AlCl_3_-to-NOTA was insignificant (*p* > 0.05). The results also showed that the RCY of the [^18^F]AlF-NOTA NHS complex decreased when the molar ratio exceeded 1:5. In view of this, the molar ratios of 1:1, 1:3, and 1:5 AlCl_3_:NOTA-pamidronic acid were chosen. The RCY of [^18^F]AlF-NOTA-pamidronic acid, determined by the radio-TLC scanner using ITLC-SG strips as adsorbents and a mobile phase of 1M ammonium acetate and acetonitrile (1:1), was above 60% for all the molar ratios prepared (Figure 9) and, thus, met the requirement to prepare [^18^F]AlF-NOTA-pamidronic acid with an acceptable RCY between 40 and 60%. The RCY of [^18^F]AlF-NOTA-pamidronic acid was highest (*M* = 95.50%, *SD* = 5.34) when the NOTA-pamidronic acid was prepared at a molar ratio of 1:1 with AlCl_3_ (*n* = 6). By contrast, increasing the molar ratio of the NOTA-pamidronic acid precursor to 1:3 and 1:5 did not further increase the formation of [^18^F]AlF-NOTA-pamidronic acid. This result was expected as the presence of excess chelator reduces RCY, as, in this case, it did with the NOTA-pamidronic acid [38].

Interestingly, the formation of [^18^F]AlF-NOTA-pamidronic acid was the second highest (*M* = 80.74%, *SD* = 5.73) when the molar ratio of the NOTA-pamidronic acid precursor was reduced to half (*n* = 6). This result suggests that even at a lower molar ratio of AlCl_3_-to-NOTA-pamidronic acid (2 µmol AlCl_3_: 1 µmol NOTA-pamidronic acid), an RCY of more than 60% can be successfully achieved. This could bring economic advantages later on when upscaling the radiolabeling of NOTA-pamidronic acid, as production costs could be reduced due to the lower amount of NOTA-pamidronic acid precursor.

**Figure 10:**
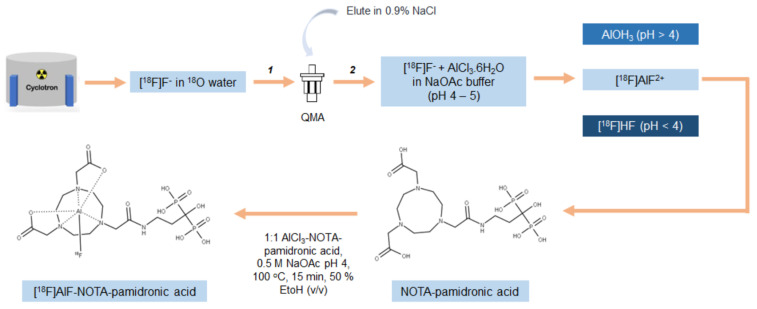
Schematic representation of the process for optimal radiolabeling conditions for [^18^F]AlF-NOTA-pamidronic acid (1: purification; 2: fractionation).

Further statistical analysis of the one-way ANOVA revealed that the difference in the percentage of the RCY (formation) of [^18^F]AlF-NOTA-pamidronic acid between the prepared molar ratios of AlCl_3_-to-NOTA-pamidronic acid was significant (*p* < 0.05). The Games–Howell post hoc analysis of the percentage of RCY of [^18^F]AlF-NOTA-pamidronic acid revealed that increasing the molar ratio of AlCl_3_-to-NOTA-pamidronic acid beyond 1:1 significantly reduced the percentage of RCY (*p* < 0.05). The difference in the percentage RCY of [^18^F]AlF-NOTA-pamidronic acid was insignificant when the molar ratio of 1:0.5 was compared to the molar ratio of 1:3, indicating that although 1 µmol (molar ratio of 1:0.5) and 6 µmol (molar ratio of 1:3) of the NOTA-pamidronic acid precursor could achieve a yield of more than 60%, a higher amount of the NOTA-pamidronic acid precursor was required (6 µmol) to approach the exact [^18^F]AlF-NOTA-pamidronic acid complexation with a molar ratio of 1:0.5 (1 µmol). The optimal radiolabeling conditions (Figure 10) that could produce [^18^F]AlF-NOTA-pamidronic acid complexation in more than 90% RCY were as follows (Table 4).

Our experimental results also confirmed that QMA-bound [^18^F]F^−^ could be eluted with 0.9% saline without the need for pH adjustment by eluting with 0.4 M KHCO_3_ followed by acetic acid [52,53]. In the early phase of the radiolabeling studies, the QMA-bound [^18^F]F^−^ was eluted with 0.4 M KHCO_3,_ and the KHCO_3_ was subsequently neutralised with acetic acid [52]. Although a one-step labeling approach (one-pot approach) was used in most previous applications, a two-step labeling approach was used in the present study: preparing the NOTA-pamidronic acid precursor in its pure form and using the NOTA-pamidronic acid as a precursor for the labeling step with aqueous [^18^F]AlF^2+^. The two-step labeling approach used in the experimental design differed slightly from that of Vogg et al. and D’Souza et al., as their approach first involved the formation of the purified [Al(OH)(NODA)] complex or peptide-aluminium complex and then the ligand exchange of [OH]^−^ for [^18^F]F^−^ [30,52]. Vogg emphasised that even with the correct pH and ethanol in the aqueous reaction medium, they could not achieve an RCY above 80% [30]. They pointed out that heating to 100 °C may have led to the thermal stability of the [Al(OH)(NODA)] complex in the presence of [^18^F]F^−^ during labeling, releasing the Al^3+^. Al^3+^ then readily combined with the [^18^F]F^−^ to form [^18^F]AlF^2+^, which, in turn, formed a low concentration of Al-free NODA complexes for reaction. Therefore, they assumed that the addition of the metal-free NODA, right from the beginning of the second labeling step, could have increased the yield to over 80% [30]. This could be the reason why an RCY of over 90% was achieved for the [^18^F]AlF-NOTA-pamidronic acid in this experiment.

In our experiment, the choice of a two-step labeling approach proved to be very applicable, although pamidronic acid could survive at higher temperatures during labeling. The metal-free NOTA-pamidronic acid was prepared in its purest form before the labeling step, with the aqueous [^18^F]AlF^2+^ formed in the first labeling step. The prepared NOTA-pamidronic acid was isolated from the crude sample, leaving the free NOTA. Free NOTA with an N_3_O_2_ donor proved to be the most stable [^18^F]AlF efficiently at higher temperatures (100–120 °C) and could interfere with the radiolabeling of NOTA-pamidronic acid if present. Therefore, instead of [^18^F]AlF-pamidronic acid, [^18^F]AlF-free NOTA moieties could also be formed.

In summary, the radiolabeling of NOTA-pamidronic acid using an aluminium-fluoride technique was straightforward and could be completed within 30 min without time-consuming drying steps and eliminating the need for high-performance liquid chromatography (HPLC) or SPE for purification. In contrast to the [^18^F]AlF labeling strategy, most ^18^F labeling strategies are tedious to perform and require multiple purifications of intermediates, resulting in a low RCY. The [^18^F]AlF labeling strategy enables the significant redesign of existing radiopharmaceuticals as replacements for ^68^Ga, as demonstrated by the many examples where [^18^F]AlF-derivatives of ^68^Ga-peptides have been developed to overcome the limitations of ^68^Ga [34]. The preliminary data suggest that ^68^Ga and [^18^F]AlF radiopharmaceuticals have similar pharmacokinetic profiles, although differences have been observed in some cases, particularly in biodistribution [54].

#### 2.2.6. Molar Activity (A_m_)

**Figure 11:**
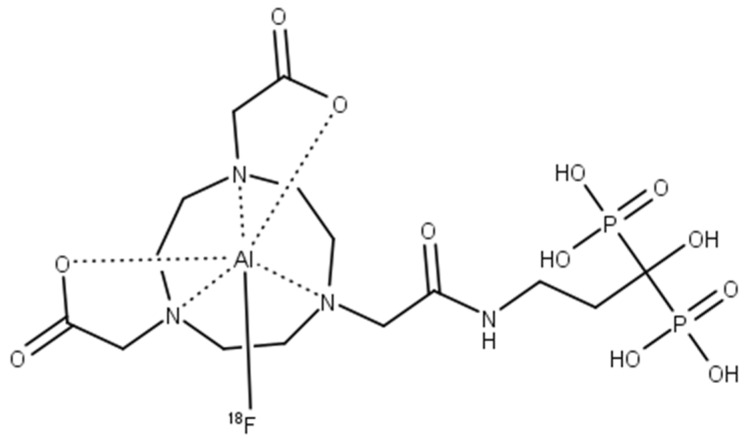
The structure of [^18^F]AlF-NOTA-pamidronic acid.

Under these conditions, [^18^F]AlF-NOTA-pamidronic acid (Figure 11) was obtained with molar activities (A_m_) of 0.024 GBq µmol^−1^ (*SD* = 0.002) at the end of the syntheses (*n* = 6)

### 2.3. Quality Control Analysis of [^18^F]AlF-NOTA-Pamidronic acid

#### 2.3.1. Radiochemical Purity (RCP) Analysis of [^18^F]AlF-NOTA-Pamidronic Acid Using RP-HPLC

**Figure 12:**
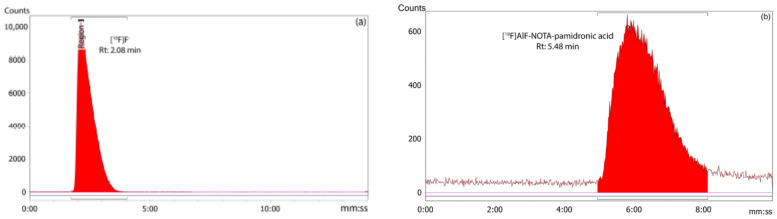
(**a**) Peak corresponding to the [^18^F]F^−^ and (**b**) [^18^F]AlF-NOTA-pamidronic acid from the radiochromatogram of RP-HPLC.

The RCP of the [^18^F]AlF-NOTA-pamidronic acid was 100%, based on the RP-HPLC analysis (*n* = 12) (Figure 12). The Rt of the [^18^F]AlF-NOTA-pamidronic acid at 5.46 min (*SD* = 0.05) was similar to the Rt of the NOTA-pamidronic acid precursor. The relative standard deviation (RSD) percentage was within 2% (*n* = 6). No free ^18^F or [^18^F]AlF^2+^ was detected in the radiochromatogram, indicating that the NOTA-pamidronic acid precursor was successfully radiolabeled with [^18^F]AlF^2+^ complexes. The unbound [^18^F]F^−^ or [^18^F]AlF^2+^ peak appeared at 2.09 min (*SD* = 0.02) under this chromatographic condition.

##### RCY Analysis of the [^18^F]AlF-NOTA-Pamidronic Acid Using r-TLC

**Figure 13:**
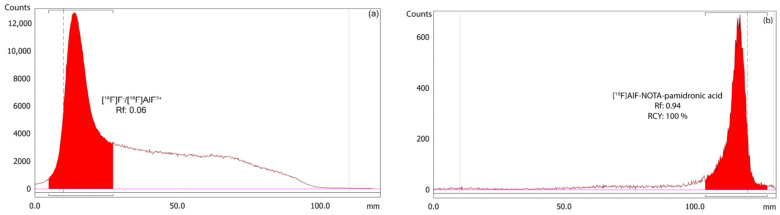
(**a**) Peak corresponding to the [^18^F]F^−^/[^18^F]AlF^2+^, and (**b**) [^18^F]AlF-NOTA-pamidronic acid from the radiochromatogram of r-TLC.

The RCY of the [^18^F]AlF-NOTA-pamidronic acid was 100%, based on an r-TLC analysis of the determined [^18^F]AlF-NOTA-pamidronic acid sample obtained when NOTA-pamidronic acid was prepared in a molar ratio of 1:1 with AlCl_3_ (*n* = 6) (Figure 13). [^18^F]AlF-NOTA-pamidronic acid was spotted onto the ITLC-SG strip at the origin, and the strip was developed to the solvent front in a solvent mixture of 1M of ammonium acetate and acetonitrile (1:1). The retention factor (Rf) of the [^18^F]AlF-NOTA-pamidronic acid, 0.91 (*SD* = 0.005), was within the acceptance criteria of Rf 0.6 to 1.0. The %RSD was within 2% (*n* = 6). No free [^18^F]F^−^ or [^18^F]AlF^2+^ was detected in the respective radiochromatogram, which is normally retained at an Rf of 0 to 0.4. Based on these findings, the Rf of [^18^F]F^−^ or [^18^F]AlF^2+^ was 0.06 (*SD* = 0.002) when unbound [^18^F]F^−^ or [^18^F]AlF^2+^ was spotted onto the ITLC-SG strip and developed under the same chromatographic conditions.

#### 2.3.2. Residual Solvents Analysis

**Figure 14:**
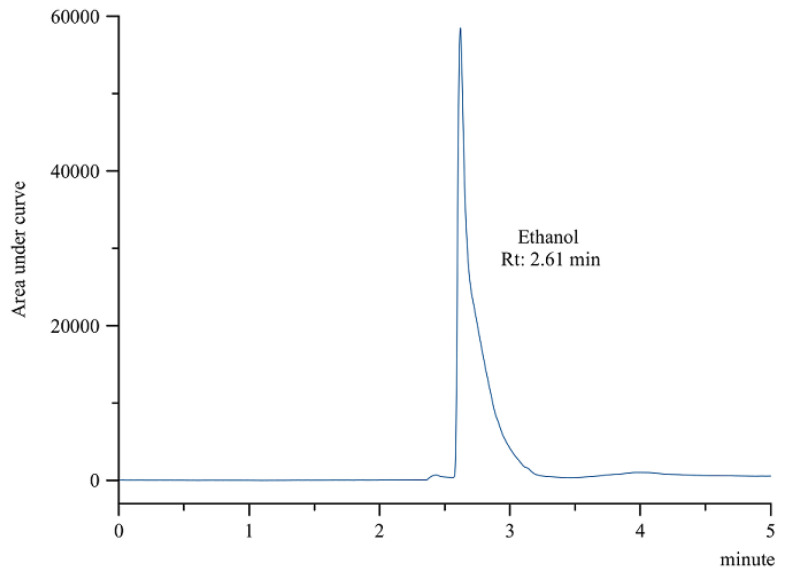
GC-FID chromatogram of ethanol peak from [^18^F]AlF-NOTA-pamidronic acid sample.

Since only ethanol 50% (*v*/*v*) was added in the radiolabeling of the [^18^F]AlF-NOTA-pamidronic acid, only ethanol was detected in the final formulation by gas chromatography (GC). The residue of ethanol was detected in the [^18^F]AlF-NOTA-pamidronic acid sample at an Rt of 2.61 min (*SD* = 0.01) (*n* = 6) (Figure 14), which was similar to the previously determined Rt of the ethanol standard solution. The %RSD was also within 2%. Using a previously prepared calibration curve, the concentration of the ethanol in the [^18^F]AlF-NOTA-pamidronic acid sample was determined to be 1.353 mg mL^−1^, which was below the threshold of 5 mg mL^−1^.

#### 2.3.3. Stability Study of [^18^F]AlF-NOTA-Pamidronic Acid

**Figure 15:**
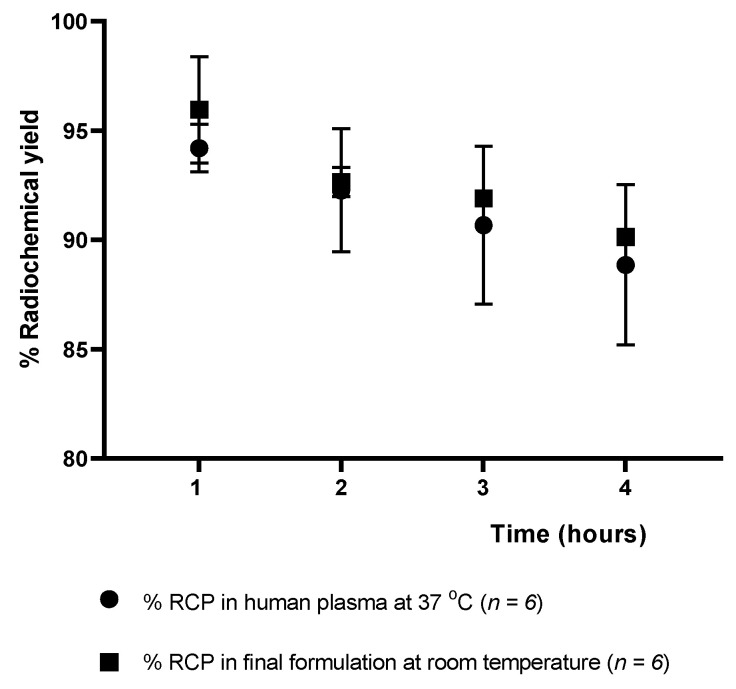
Stability of the [^18^F]AlF-NOTA-pamidronic acid in the final formulation and in human plasma up to 4 h [mean (SEM)].

The stability of the [^18^F]AlF-NOTA-pamidronic acid was determined at 1, 2, 3, and 4 h after radiolabeling (Figure 15), verifying that the radiochemical purity remained higher than 90% for 4 h in the final formulation vial even without the addition of ascorbic acid as a radioprotective agent. The result was consistent with the fact that the NOTA ligand, in this case, NOTA-pamidronic acid, is known to form stable complexes with the Al^3+^ (AlCl_3_) [51]. The RCP of [^18^F]AlF-NOTA-pamidronic acid was determined to be 90.15% (*SD* = 0.06) (*n* = 6) after 4 h of radiolabeling.

The RCP of the [^18^F]AlF-NOTA-pamidronic acid was 90.67% (*SD* = 3.62) (*n* = 6) after 3 h of radiolabeling, indicating that the requirement of more than 90% of the radioactivity was in the form of the [^18^F]AlF-NOTA-pamidronic acid was met in human plasma at 37 °C (3 h). In contrast to other studies that also used NOTA as a chelator or the Al-F technique for radiopharmaceutical development, the corresponding radiopharmaceutical was no longer stable in human plasma from 60 to 90 min onwards [35,55].

A possible explanation could be that the radioactivity of [^18^F]F^−^ used in this experiment was lower (less than 30 MBq) than in other studies where a higher radioactivity (more than 400 MBq) was used, even with the same NOTA chelator, which could have contributed to the instability of this radiopharmaceutical in the human plasma [35]. For this reason, special care should be taken in the future when upscaling production to prevent the possible instability of [^18^F]AlF-NOTA-pamidronic acid in human plasma. Based on this information, it should be considered that images of PET should be acquired within 30–60 min of the intravenous injection if used for preclinical or clinical imaging in the future.

In summary, [^18^F]AlF-NOTA-pamidronic acid, which was prepared in two consecutive runs, met all the quality control analysis requirements for the appearance, pH, radiochemical purity, and organic solvent analysis for the radiopharmaceutical. [^18^F]AlF-NOTA-pamidronic acid was also stable in the final formulation and in human plasma at 37 °C (4 h). Table 5 below summarises the quality control analyses performed on the [^18^F]AlF-NOTA-pamidronic acid sample with the respective acceptance criteria and observed value.

### 2.4. In Vitro Binding Studies of [^18^F]AlF-NOTA-Pamidronic Acid

#### 2.4.1. In Vitro Bone Binding Assay Using Hydroxyapatite (HA)

**Figure 16:**
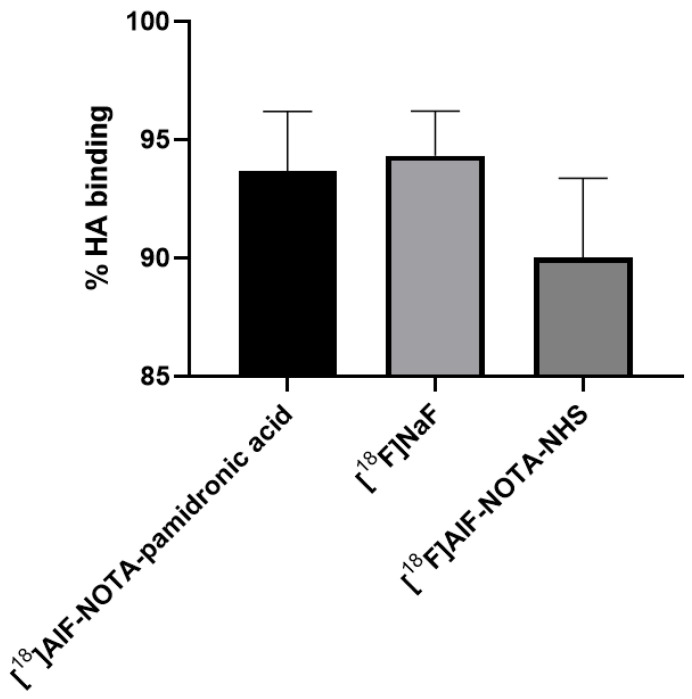
The % bone binding assay experimented using HA for [^18^F]AlF-NOTA-pamidronic, [^18^F]NaF and [^18^F]AlF-NOTA (*n* = 3) [mean (SEM)].

The in vitro studies in this experiment were performed with synthetic HA using the method described by Meckel et al. [27]. The HA binding assay (Figure 16) showed a higher binding of [^18^F]NaF (*M* = 94.31%, *SD* = 3.28), followed by [^18^F]AlF-NOTA-pamidronic acid (*M* = 93.68%, *SD* = 4.35) and [^18^F]AlF-NOTA (*M* = 90.04%, *SD* = 5.77) (*n* = 3). Statistical analysis of the one-way ANOVA revealed that the difference in the percentage binding of HA was insignificant for all three agents (*p* > 0.05). The results showed that a higher HA binding assay of more than 90% was achieved with the ^18^F derivative than when the ^68^Ga derivatives were used [23,27,56,57,58]. The in vitro HA binding assay recorded the highest value of 91% only with ^68^Ga-DOTA-pamidronic acid, while the majority ranged from 70 to 85%.

This result was to some extent expected, as both [^18^F]AlF-NOTA-pamidronic acid and [^18^F]NaF show a high binding affinity to HA. The difference in% of HA binding was only 0.63% between the two. The result was similar to that of Keeling et al., who compared the in vitro bone binding between [^68^Ga]Ga-THP-Pamidronate and [^18^F]NaF [23]. [^18^F]NaF binds to HA, displacing the hydroxyl groups within the HA lattice for [^18^F]F^−^ [23]. This could be the reason why the binding of HA was also observed in [^18^F]AlF-NOTA, as it possibly behaves similar to [^18^F]F^−^ when NOTA is hydrolysed in 0.9% NaCl solutions and becomes an [^18^F]AlF^2+^ complex that can be incorporated into the HA surface [59].

The results showed that [^18^F]AlF-NOTA-pamidronic acid was also sensitive to the presence of HA and was able to bind to HA similar to [^18^F]NaF. However, the experiment did not provide further evidence for the specificity of [^18^F]AlF-NOTA-pamidronic acid and [^18^F]NaF. Therefore, the in vitro cellular uptake studies conducted in the following section demonstrate the specificity of the uptake of [^18^F]AlF-NOTA-pamidronic acid, [^18^F]NaF, and [^18^F]AlF-NOTA for the cell lines used.

#### 2.4.2. In Vitro Cellular Uptake Studies

**Figure 17:**
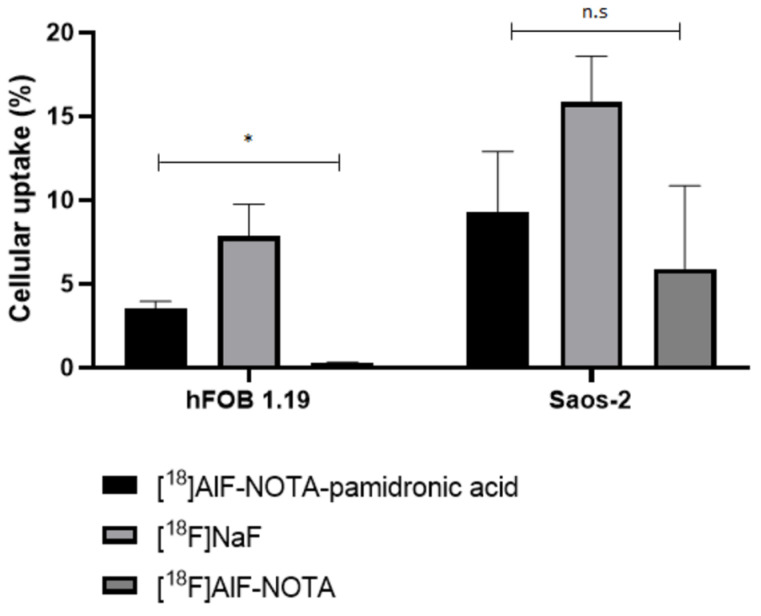
Cellular uptake of the [^18^F]AlF-NOTA-pamidronic acid, [^18^F]NaF and [^18^F]AlF-NOTA in hFOB 1.19 and Saos-2 cells (*n* = 3) at time point 30 min (* *p* < 0.05, n.s: non-significant) [mean (SEM)].

In this section, the specificity for the uptake of [^18^F]AlF-NOTA-pamidronic acid, [^18^F]NaF, and [^18^F]AlF-NOTA for the normal human osteoblast cell line (hFOB 1.19) and human osteosarcoma cell lines (Saos-2) is investigated. The radioactivity that accumulated on the surface in both cell lines after 30 min of incubation with [^18^F]AlF-NOTA-pamidronic acid, [^18^F]NaF, and [^18^F]AlF-NOTA was counted using a gamma counter (*n* = 3). The result showed that the uptake of [^18^F]NaF was higher in both cell lines, followed by [^18^F]AlF-NOTA-pamidronic acid, the compound of interest for this study (Figure 17). This mirrors the results from the in vitro HA binding assay.

In the hFOB 1.19 cell lines, the [^18^F]NaF uptake was 7.84% (*SD =* 3.31), followed by an [^18^F]AlF-NOTA-pamidronic acid uptake of 3.52% (*SD* = 0.76). The observed cellular uptake of [^18^F]AlF-NOTA for hFOB 1.19 was almost negligible at 0.28% (*SD* = 0.07). The cellular uptake of [^18^F]NaF was 2-fold higher than the uptake of [^18^F]AlF-NOTA-pamidronic acid in the hFOB 1.19 cell lines. The cellular uptake of [^18^F]AlF-NOTA was very low, possibly due to the presence of a bifunctional chelator of NOTA that limited the surface binding of [^18^F]AlF-NOTA to the hFOB 1.19 cell lines. On the other hand, this shows that NOTA formed stable complexes with [^18^F]AlF^2+^, and the [^18^F]AlF-NOTA complex in this experiment was not further transchelated to [^18^F]AlF^2+^ when performed in vitro [51].

Further statistical analysis of the one-way ANOVA revealed that the difference in cellular uptake was significant for all three radiopharmaceuticals (*p* < 0.05). This result was somewhat expected, as [^18^F]NaF has been used primarily for the early diagnosis and monitoring of bones in PET [59]. Although the result shows that the cellular uptake of [^18^F]AlF-NOTA-pamidronic acid in hFOB 1.19 was 2-fold lower compared to [^18^F]NaF, it was nevertheless demonstrated that [^18^F]AlF-NOTA-pamidronic acid can bind to normal osteoblast cells. The evidence supports the concept that [^18^F]AlF-NOTA-pamidronic acid has the potential to be used for PET bone imaging.

Although [^18^F]NaF showed higher cellular uptake in the Saos-2 cell lines (*M* = 15.84%, *SD* = 4.78), the difference in the cellular uptake compared to [^18^F]AlF-NOTA-pamidronic acid (*M* = 9.28%, *SD* = 6.25) was not significant when further statistically analysed using a one-way ANOVA (*p* > 0.05) (Figure 16). The amount of accumulated radioactivity between [^18^F]NaF and [^18^F]AlF-NOTA-pamidronic acid was not 2-fold higher than that previously observed in hFOB 1.19 cell lines.

This finding literally suggests that the compound investigated in this study, [^18^F]AlF-NOTA-pamidronic acid, tends to be more specific than [^18^F]NaF, in the sense that the cellular uptake of [^18^F]AlF-NOTA-pamidronic acid in the Saos-2 cell lines was higher than the cellular uptake of [^18^F]AlF-NOTA-pamidronic acid in the hFOB 1.19 cell lines. Furthermore, the difference in cellular uptake of [^18^F]AlF-NOTA-pamidronic acid and [^18^F]NaF in the Saos-2 cell lines was insignificant, whereas the difference in the cellular uptake of [^18^F]AlF-NOTA-pamidronic acid and [^18^F]NaF in hFOB 1.19 cell lines was significant.

Nevertheless, the study does not attempt to mislead the audience by simply drawing a conclusion about the specificity of [^18^F]AlF-NOTA-pamidronic acid in the Saos-2 cell lines compared to [^18^F]NaF. The focus of this study is on the potential of [^18^F]AlF-NOTA-pamidronic acid in PET bone imaging. There may be an explanation for the insignificant difference in the cellular uptake of [^18^F]AlF-NOTA-pamidronic acid and [^18^F]NaF observed in the Saos-2 cell lines. Although the Saos-2 cell line has several osteoblastic features, further studies have shown that osteosarcomas histologically express two other common features, namely chondroblastic and fibroblastic [3,60,61]. Chondroblasts contribute to the formation of cartilage, while fibroblasts form connective tissues that support and connect other tissues or organs in the body [60]. Since the osteoblastic, chondroblastic, and fibroblastic subtypes are predominantly expressed in osteosarcomas, it is possible that the compound investigated in this study, [^18^F]AlF-NOTA-pamidronic acid, could provide additional information that [^18^F]NaF could not.

Based on this evidence, this study has proved that the concerns of Bastawrous et al. were valid, as the uptake of [^18^F]NaF is non-cancer-specific, and non-malignant cells can also show an uptake, as observed in the case of the significant cellular uptake in normal human osteoblasts (hFOB 1.19). This is consistent with reports of a higher number of false-positive interpretations with [^18^F]NaF [19]. Nevertheless, this study concludes that the investigated compound [^18^F]AlF-NOTA-pamidronic acid has the potential to be used in PET bone imaging and is substantially able to provide additional information that [^18^F]NaF could not provide in the case of osteosarcoma disease.

#### 2.4.3. Limitation of the Study

This study reports only the bone binding affinity of the [^18^F]AlF-NOTA-pamidronic acid on its in vitro binding affinity to HA and in vitro cellular uptake to demonstrate the uptake of [^18^F]AlF-NOTA-pamidronic acid. The in vitro cellular uptake studies were only performed at a one-time point (30 min) due to the distance between the laboratory and the gamma counter facility for counting. The comparison of both studies with [^18^F]NaF and [^18^F]AlF-NOTA have only been discussed superficially and is beyond the scope of this study. The comparison of the in vitro binding affinity studies between [^18^F]AlF-NOTA-pamidronic acid and [^18^F]NaF can be a comprehensive study for future endeavours.

## 3. Materials and Methods

### 3.1. Materials

Pamidronic acid (C_3_H_11_NO_7_P_2_) (Santa Cruz Biotechnology, Dallas, TX, USA), NOTA-NHS ester (C_6_H_24_N_4_O_8_) (CheMatech, Dijon, France), ^18^F radionuclide (National Cancer Institute, Putrajaya, Malaysia), synthetic hydroxyapatite (HA) (Sigma Aldrich, St. Louis, Mo, USA), osteoblast cell line (hFOB 1.19) (ATCC, Manassas, VA, USA), osteosarcoma cell line (Saos-2) (ATCC, USA), human plasma (Department of Medical Microbiology, HPUPM, Serdang, Malaysia), dimethylformamide (DMF), 0.1% trifluoroacetic acid (C_2_HF_3_O_2_) >99.9%, aluminium chloride hexahydrate (AlCl_3_.6H_2_O), triethylamine (C_6_H_15_N) >99% (Sigma Aldrich, USA), dimethyl sulfoxide (DMSO), ammonium acetate (NH_4_CH_3_CO_2_) (Sigma Aldrich, USA), acetic acid (CH_3_COOH), acetonitrile (Supelco, Bellefonte, USA), formic acid (CH_2_O_2_), ethanol, sodium acetate (C_2_H_3_NaO_2_) (Merck, Kenilworth, USA), water for injection (WFI), 0.9% sodium chloride (B. Braun, USA), Geneticin^®^ (G418 sulfate), penicillin streptomycin (ThermoFisher Scientific, Waltham, MA, USA), Foetal bovine serum, Dulbecco’s phosphate-buffered saline (PBS), Trypsin/EDTA, sterile DMSO (ATCC, USA), Instant Thin Layer Chromatography-Silica Gel (ITLC-SG) (Agilent, Santa Clara, CA, USA).

### 3.2. Methods

#### 3.2.1. Preparation, Validation, and Isolation of NOTA-Pamidronic Acid Precursor

In general, the preparation of the NOTA-pamidronic acid precursor involves the dilution of the weighed pamidronic acid in 2.5 mL of deionised water. The mixture was vortexed until completely dissolved. A careful adjustment of the pH to 8 followed using pH indicator strips during the addition of 30 µL TEA. Next, NOTA-NHS was weighed and dissolved in 1.5 mL DMF before about 50 µL fractions of the NOTA-NHS solution were added to the dissolved pamidronic acid (650 µL) and prepared in triplicate (*n* = 3).

**Figure 18:**
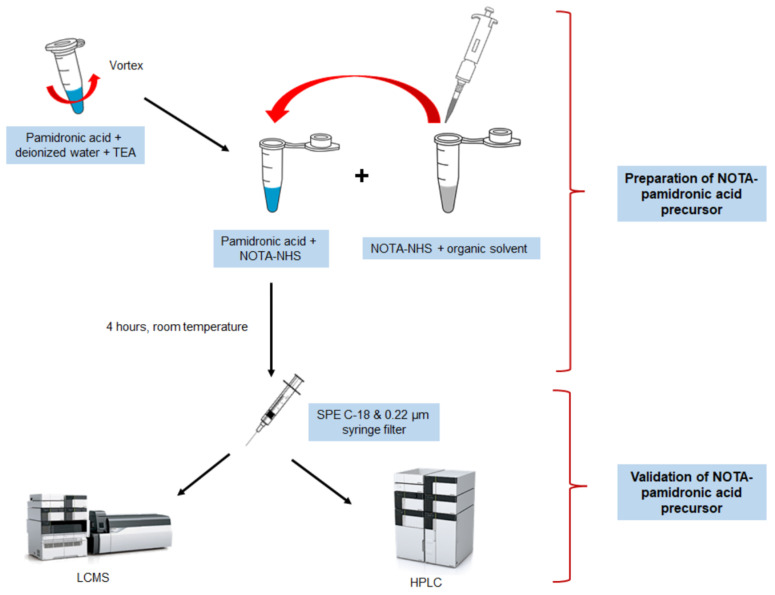
Workflow illustration of the NOTA-pamidronic acid preparation process.

The pH of the reaction was monitored every hour with a pH indicator strip. The reaction condition should be neutral to slightly basic [37]. After 4 h, and prior to validation, the crude NOTA-pamidronic acid was filtered by SPE using a Sep-Pak C18 Plus Light cartridge (Waters, Milford, MA, USA) and a 0.22 µm nylon syringe filter (PhenexTM-NY, USA) to remove organic impurities. A series of experiments were carried out varying the molar ratio of the pamidronic acid: NOTA-NHS by preparing both materials according to Table 6. Figure 18 shows the workflow for the preparation of the NOTA-pamidronic acid precursor.

#### 3.2.2. Validation of NOTA-Pamidronic Acid Using LC-MS Analysis

LC-MS analysis was performed using an LC Dionex Ultimate 3000 (Thermo Fisher Scientific, Waltham, MA, USA) equipped with an autosampler, quaternary pump, column compartment, UV, and diode-array detector (PDA). Chromatographic analysis was performed using a Synergi Hydro-RP 2.5 µm, 50 × 2.1 mm column (Phenomenex, Torrance, CA, USA). The column temperature was 30 °C throughout the analysis. Since the sample compounds were polar, reversed-phase chromatography with ion suppression was used in this analysis. The mobile phase was 0.1% formic acid in the water, while the flow rate was set at 0.3 mL min^−1^. Approximately 10 µL of the sample was injected for each LC-MS analysis. The ionisation mode was set to a negative ion mode with a collision energy of 30 eV. The *m*/*z* scan was performed from *m*/*z* 50 to 750. The chromatogram and mass spectrum (precursor ion and isotopic abundance) were analysed using a Thermo Xcalibur 4.2.47. The percentage yield of the NOTA-pamidronic acid precursor and free pamidronic acid was recorded and calculated from the chromatogram.

#### 3.2.3. Isolation of the NOTA-Pamidronic Acid Fraction from the Crude Sample

The NOTA-pamidronic acid fraction was isolated and collected from the crude product using an RP-HPLC (Agilent 1200, USA) equipped with a fraction collector. The isolation of the NOTA-pamidronic acid fractions was carried out under optimal chromatographic conditions using a Synergi 4µ (C18, polar endcapped) column, 0.1% trifluoroacetic acid (TFA) in water as a mobile phase at 0.5 mL min^−1^ with detection at 220 nm. A sample injection volume of 10 µL was manually injected using a microlitre syringe (Glass capillary, Hamilton). The fractions corresponding to each peak in the chromatogram–free pamidronic acid, NOTA-pamidronic acid, and free NOTA were repeatedly collected for 5 mL in separate vials. However, only the collected NOTA-pamidronic acid fractions were re-analysed using analytical RP-HPLC before being sent for freeze-drying.

#### 3.2.4. Preparation of [^18^F]AlF^2+^ Complexes

Using the highest fraction of [^18^F]F^−^ radioactivity previously collected in the (PCR) tube, 10 µL of the [^18^F]F^−^ with radioactivity of ~5 to 10 MBq was added to a 0.2 mL vial containing 10 µL of 1 mM AlCl_3_ in 0.5 M sodium acetate buffer solution at pH 4. The reaction mixture was thoroughly mixed and incubated at room temperature for 10 min to allow the formation of [^18^F]AlF^2+^ complexes.

#### 3.2.5. Quality Control Analysis for [^18^F]AlF-NOTA-Pamidronic Acid

[^18^F]AlF-NOTA-pamidronic acid was analysed for the appearance of pH, RCP, RCY, residual solvent, and in vitro stability study. Chromatographic separation for the residual solvent analysis was performed in accordance with Hassan et al. [62].

#### 3.2.6. In Vitro Stability Study of [^18^F]AlF-NOTA-Pamidronic Acid

The stability study of the [^18^F]AlF-NOTA-pamidronic acid in normal saline (0.9% NaCl) and in vitro human plasma was studied at room temperature after 1, 2, 3, and 4 h using the r-TLC method. The stability of the [^18^F]AlF-NOTA-pamidronic acid (100 µL) was tested by incubating 900 µL of human plasma at 37 °C for up to 4 h. After 1-, 2-, 3-, and 4-h incubation, about 200 µL were precipitated with 200 µL of ethanol and centrifuged at 12,000 rpm for 5 min before being analysed by the r-TLC method [55,63].

#### 3.2.7. In Vitro Binding Studies of [^18^F]AlF-NOTA-Pamidronic Acid

##### In Vitro Bone Binding Assay Using Hydroxyapatite

A total amount of 50 µL of radiolabeled [^18^F]AlF-NOTA-pamidronic acid was added to the prepared HA assay and incubated for 10 min. After incubation, the supernatant was carefully removed with the Pasteur pipette, leaving HA in the centrifuge tube. Next, 500 µL of normal saline was added and then centrifuged at 3000 rpm. This time, the supernatant was transferred to another centrifuge tube (B), leaving the retained [^18^F]AlF-NOTA-pamidronic acid on HA (A). This experiment was performed simultaneously with [^18^F]NaF and [^18^F]AlF-NOTA. The bone binding assay was determined with a gamma counter and calculated with the following Equation (1):

(1) $$\%\;bone\;binding\;assay=\frac{fraction\;A}{fraction\;A+fraction\;B\;}\times 100\%$$

##### In Vitro Cellular Uptake Studies

Cell lines hFOB 1.19 and Saos-2 were seeded in 12-well plates at a density of 1 × 10^5^ cells/well 2 days prior to the cellular uptake studies. Complete culture media (CCM) was freshly replaced on the day of the experiment. The cells were then incubated with [^18^F]AlF-NOTA-pamidronic acid (90–140 kBq/well), which was adjusted to a final volume of 0.5 mL with CCM. After 30 min, the supernatant was collected, and the cells were washed twice with PBS. The radioactivity that accumulated on the surface of both cell lines was measured with a gamma counter. The radioactive medium and the collected PBS were defined as C_out_. Finally, the cells were harvested with trypsin and washed again twice with PBS. The radioactivity of the harvested cells and PBS was defined as C_in_. The cellular uptake rate was calculated using the following formula (Equation (2)). The above steps were repeated for [^18^F]NaF and [^18^F]AlF-NOTA. Each of the cell lines was prepared in triplicate for each of the radiopharmaceuticals used (*n* = 3).

(2) $$Cellular\;uptake=\frac{Cin}{\left(Cin+Cout\right)\;}\times 100\%$$

The human osteoblast cell line hFOB 1.19 (ATCC^®^ CRL-11372^TM^) was acquired from the Animal Type Culture Collection (ATCC, Manassas, VA, USA). The human osteosarcoma cell line, Saos-2 (ATCC HTB-85^TM^), was obtained from Dr. Azuraidi Osman at the Department of Cell and Molecular Biology, Faculty of Biotechnology and Biomolecular Sciences, UPM. The in vitro study was performed with the approval of the Institutional Biosafety and Biosecurity Committee (IBBC), Universiti Putra Malaysia (UPM/IBBC/NGMO/2021/R004).

## 4. Conclusions

[^18^F]AlF-NOTA-pamidronic acid was successfully prepared and optimised by exploiting the fluorophilic nature of aluminium to form a stable complex cation [^18^F]AlF^2+^, followed by coordination with the NOTA-pamidronic acid moiety. The preliminary in vitro results demonstrate that the [^18^F]AlF-NOTA-pamidronic acid can potentially be used for PET bone imaging. One of the most intriguing results was to witness its specificity when a higher cellular uptake of [^18^F]AlF-NOTA-pamidronic acid was observed in the Saos-2 cell lines than in the hFOB 1.19 cell lines. These preliminary results suggest that a comprehensive preclinical study of [^18^F]AlF-NOTA-pamidronic acid is required before it can be moved into clinical research.

## Figures and Tables

**Table 1:** Percentage yield of NOTA-pamidronic acid.

Pamidronic Acid: NOTA Molar Ratio	Peak Area (Average, *n* = 3)		% Yield
	Free Pamidronic Acid	NOTA-Pamidronic Acid	
5:1	3,409,680,392	1,084,594,404	24.13
10:1	5,242,116,420	1,155,377,273	18.06
15:1	8,343,559,233	1,243,259,017	12.97

**Table 2:** The calculated *m*/*z* and obtained *m*/*z* of pamidronic acid, NOTA-pamidronic acid, and NOTA by product; ESI negative mode *m*/*z* vale [M-H]^−^ (*n* = 3).

Compound	[M-H]^−^ Calculated *m*/*z*	[M-H]^−^ Obtained *m*/*z*	Relative Error (ppm)
Pamidronic acid	233.9932	233.9934	0.9972
NOTA-pamidronic acid	519.1263	519.1265	0.4495
NOTA	302.1358	302.1358	0.0000

**Table 3:** The relative error (ppm) and RDBE for each fragment produced from the MS–MS analysis.

Obtained *m*/*z*	Exact *m*/*z* (Predicted)	Relative Error (ppm)	RDBE	Molecular Formula
519.1265	519.1263	0.3853	4.5	C_15_H_29_N_4_O_12_P_2_
501.1159	501.1157	0.3991	5.5	C_15_H_27_N_4_O_11_P_2_
437.1442	437.1443	0.2288	5.5	C_15_H_26_N_4_O_9_P_1_
393.1546	393.1545	0.2544	4.5	C_14_H_26_N_4_O_7_P_1_
283.1772	283.1776	1.4125	4.5	C_13_H_23_N_4_O_3_
152.0108	152.0118	6.5784	1.5	C_3_H_7_NO_4_P_1_
142.9294	142.9299	3.4982	1.5	H_1_O_5_P_2_
134.9841	134.9847	4.4449	2.5	C_3_H_4_O_4_P_1_

**Table 4:** Optimal radiolabeling conditions.

Variables	Optimal Conditions
AlCl_3_ concentration	2 mM
AlCl_3_-to-NOTA-pamidronic acid molar ratio	1:1 (2 µmol NOTA-pamidronic acid)
Reaction temperature	100 °C
Reaction time	15 min
Organic solvent	Ethanol 50% (*v*/*v*)

**Table 5:** Quality control analysis of [^18^F]AlF-NOTA-pamidronic acid (*n* = 6).

Quality Control Analysis	Acceptance Criteria	[^18^F]AlF-NOTA-Pamidronic Acid
Appearance	Clear, colourless and free of particles	Verified
pH	4 to 8	7
RCP (HPLC)	≥90%	100%
RCY (ITLC-SG)	90%	95%
Organic solvent: ethanol (GC)	≤5 mg mL^−1^	1.353 mL^−1^

**Table 6:** Pamidronic acid and NOTA-NHS molar ratio.

Pamidronic Acid (mg)	Pamidronic Acid (mM)	NOTA-NHS (mM)	Pamidronic Acid: NOTA-NHS Molar Ratio
5.875	10	2	5:1
11.750	20	2	10:1
17.625	30	2	15:1

## Data Availability

The datasets used and/or analysed during the current study are available from the corresponding author upon reasonable request.
